# Acute Effects on the Human Peripheral Blood Transcriptome of Decompression Sickness Secondary to Scuba Diving

**DOI:** 10.3389/fphys.2021.660402

**Published:** 2021-06-10

**Authors:** Kurt Magri, Ingrid Eftedal, Vanessa Petroni Magri, Lyubisa Matity, Charles Paul Azzopardi, Stephen Muscat, Nikolai Paul Pace

**Affiliations:** ^1^Hyperbaric Unit, Department of Medicine, Mater Dei Hospital, Msida, Malta; ^2^Department of Circulation and Medical Imaging, Faculty of Medicine and Health Sciences, NTNU Norwegian University of Science and Technology, Trondheim, Norway; ^3^Faculty of Nursing and Health Sciences, Nord University, Bodø, Norway; ^4^Department of Clinical Pharmacology and Therapeutics, Faculty of Medicine and Surgery, University of Malta, Msida, Malta; ^5^Centre for Molecular Medicine and Biobanking, Faculty of Medicine and Surgery, University of Malta, Msida, Malta

**Keywords:** decompression sickness, decompression illness, scuba diving, transcriptome, leukocyte gene expression, myeloid cell, immediate early genes

## Abstract

Decompression sickness (DCS) develops due to inert gas bubble formation in bodily tissues and in the circulation, leading to a wide range of potentially serious clinical manifestations. Its pathophysiology remains incompletely understood. In this study, we aim to explore changes in the human leukocyte transcriptome in divers with DCS compared to closely matched unaffected controls after uneventful diving. Cases (*n* = 7) were divers developing the typical cutis marmorata rash after diving with a confirmed clinical diagnosis of DCS. Controls (*n* = 6) were healthy divers who surfaced from a ≥25 msw dive without decompression violation or evidence of DCS. Blood was sampled at two separate time points—within 8 h of dive completion and 40–44 h later. Transcriptome analysis by RNA-Sequencing followed by bioinformatic analysis was carried out to identify differentially expressed genes and relate their function to biological pathways. In DCS cases, we identified enrichment of transcripts involved in acute inflammation, activation of innate immunity and free radical scavenging pathways, with specific upregulation of transcripts related to neutrophil function and degranulation. DCS-induced transcriptomic events were reversed at the second time point following exposure to hyperbaric oxygen. The observed changes are consistent with findings from animal models of DCS and highlight a continuum between the responses elicited by uneventful diving and diving complicated by DCS. This study sheds light on the inflammatory pathophysiology of DCS and the associated immune response. Such data may potentially be valuable in the search for novel treatments targeting this disease.

## Introduction

Decompression sickness (DCS) is a potentially fatal condition usually observed after scuba diving. It involves bubble formation in blood and tissues from dissolved inert gas (usually nitrogen or helium), secondary to a decrease in ambient pressure (decompression). Its manifestations range from a mild illness to a rapidly life-threatening one. One subtype of DCS presents with cutaneous manifestations, termed cutis marmorata. Cutis marmorata manifesting after diving is pathognomonic of DCS. It consists of a patchy, geographical rash usually crossing the midline, commonly involving fatty tissues such as the abdomen, breasts, buttocks, thighs, but also the torso and back. It is well-established that this subtype of DCS is associated with arterialization of bubbles via intracardiac right-to-left shunting of venous gas emboli, most commonly due to the presence of a patent foramen ovale (PFO) (Wilmshurst, 2015). Another potential mechanism is through right-to-left shunting via intrapulmonary arteriovenous anastomoses (IPAVA) (Madden et al., 2015). This subtype of DCS is therefore distinct from musculoskeletal DCS, which is thought to be due to the presence of *in situ* autochthonous bubble formation (Edmonds et al., 2015).

The standard treatment for DCS involves oxygen delivered at an elevated ambient pressure, hyperbaric oxygen (HBO). Following hyperbaric treatment, complete resolution of symptoms occurs in 80–90% of cases (Edmonds et al., 2015). Yet, victims may suffer from long-term sequelae. Of note, DCS involving the spinal cord is challenging to treat. It may result in permanent paraplegia or paraparesis, together with bladder dysfunction with or without incontinence and sexual dysfunction (Tournebise et al., 1995). The search for new treatments for DCS as adjuncts to HBO and fluid resuscitation has so far been largely unsuccessful, with only weak evidence encouraging the use of lidocaine and the non-steroidal anti-inflammatory drug tenoxicam (Bennett et al., 2012; Edmonds et al., 2015).

Transcriptomic studies provide an opportunity to explore the pathophysiologic pathways and the underlying transcriptional mechanisms that drive disease. Potentially, this can serve as a stepping-stone toward the identification of novel biomarkers or druggable targets. Gene expression studies in humans have focused on cohorts of divers completing uneventful free diving or scuba diving. Eftedal et al. (2013) characterized the peripheral blood leukocyte transcriptome in healthy, experienced human scuba divers, and demonstrated upregulation of transcripts associated with apoptosis, inflammation, and the innate immune responses. Diving-induced leukocyte-specific shifts in transcriptional patterns were described, with downregulation of genes expressed by CD8+ T-lymphocytes and natural killer cells, and upregulation of genes expressed by neutrophils, monocytes, and macrophages (Eftedal et al., 2013). Human studies have demonstrated increased expression of antioxidant enzymes in healthy uneventful scuba diving, including upregulation of catalase (*CAT*), superoxide dismutase 1 (*SOD1*), and glutathione synthetase (*GSS*) (Kiboub et al., 2018; Perović et al., 2018). Additionally, diving triggers the development of a systemic proinflammatory state through altered regulation of genes in immune pathways (Tillmans et al., 2019; Mrakic-Sposta et al., 2020). Similarly, freediving elicits changes in the leukocyte transcriptome, with an increase in neutrophil granulocytes and a decrease of cytotoxic lymphocytes possibly driven by hypoxia (Eftedal et al., 2016).

Animal models have also demonstrated upregulation of proinflammatory signaling molecules in DCS (Bigley et al., 2008; Liu et al., 2014; Wang H.T. et al., 2015). Both uneventful diving and DCS trigger changes in the peripheral blood transcriptome, and distinguishing physiological responses from pathological changes is a major challenge. To the best of our knowledge, no study has evaluated a DCS-induced transcriptomic signature in humans. In this study, we aim to explore the evolution of leukocyte gene expression in human subjects with DCS compared to closely matched divers after uneventful diving using a hypothesis-free RNA sequencing approach.

## Materials and Methods

### Ethics Statement

This study was approved by the institutional ethics review board of the University of Malta (FRECMDS_1718_058). All subjects gave written informed consent for their participation in the study and for genetic analysis. The study protocol is in compliance with the Declaration of Helsinki.

### Patient Selection and Inclusion Criteria

Recruitment of DCS cases (*n* = 7) and controls (*n* = 6) was carried out by a diving medicine physician. Details of specific inclusion and exclusion criteria are presented in Supplementary Table 1. Briefly, the control group consisted of scuba divers performing recreational diving with local diving clubs. Diving club members received an invitation to participate on a voluntary basis. Divers satisfying the inclusion and exclusion criteria were consented, recruited, and sampled at Mater Dei Hospital, Malta. DCS cases were included in this study if they presented with the typical cutis marmorata rash within 8 h of surfacing from an underwater dive. Control subjects were divers who surfaced from a deep dive (≥25 msw) without dive table violation and without any symptoms suggestive of DCS as ascertained by a diving medicine physician. Specific exclusion criteria included (a) age < 18 years (b) self-reported consumption of alcohol and/or strenuous physical activity before T2 (c) symptoms suggestive of delayed DCS presentation in controls at T2 d) acute life-threatening clinical complications or death within 72 h of surfacing. The Francis and Smith classification for dysbaric illness was recorded for each subject (Francis and Smith, 1991). All study subjects were unrelated and of Caucasian ethnicity, and none suffered from diabetes mellitus, hypertension, asthma, ischemic heart disease, or congestive heart failure at the time of sampling.

Hempleman’s stress index (*Q* = *P*√*t*) was computed for each dive in both groups, where *Q* = exposure index (ATA.min^0.5^), *P* = maximum depth in atmospheres absolute (ATA), and *t* = total dive time in minutes (Hempleman, 1952). This exposure index has been used to limit commercial diving in previous studies (Hugon et al., 2018). Additionally, the gas burden as per the Francis and Smith classification of dysbaric illness was recorded (Mathieu, 2006). According to this classification, gas burden is defined as—I. Low (e.g., within prescribed no decompression limits), II. Medium (e.g., a dive requiring decompression stops prior to surfacing) and III. High (e.g., violation of prescribed decompression requirements).

For both DCS cases and controls, whole blood was sampled at two time points, T1—within 8 h of surfacing from diving and T2—at 40–44 h after surfacing. At T1, controls received instructions to follow with regards to behavior/lifestyle in the period prior to the second sampling time. This included fasting for 10 h prior to the second sampling time, avoiding caffeine and alcohol intake and avoiding strenuous exercise. Identical instructions were provided to recruited DCS cases to follow prior to T2. All DCS cases received emergency HBO as per United States Navy Treatment Table Six between T1 and T2.

### Data Collection

For all participants, information on dive profile (maximum depth, bottom time, total time of dive, gas mix), the number of dives within the previous 7 days, and previous history of DCS was recorded. Additionally, age, gender, body mass index, smoking status, past medical history, drug history, illicit substance use, caffeine intake on the day of the dive and alcohol during the 24 h prior to the first dive of the day were also recorded.

### Sample Processing and RNA Sequencing

For RNA isolation, 2.5 mL of whole blood was collected in a PAXgene^®^ Blood RNA Tube (PreAnalytiX, Qiagen/BD) from DCS cases and controls at both T1 and T2. The samples were frozen within 24 h and stored at -80°C till further use. RNA was extracted using the PAXgene^®^ blood kit (PreAnalytiX, Qiagen/BD) and quantified by 260/280 nm absorbance using UV-spectrophotometry (Nanodrop^®^, Thermo Fisher Scientific). The quality of RNA was evaluated by RNA Integrity Number (RIN) determination using the RNA6000 Nano protocol on an Agilent 2100 Bioanalyzer system (Agilent, United States). The RIN values for samples undergoing transcriptome analysis ranged from 7.8 to 9.3. Depletion of alpha and beta globin mRNA was carried out using the GLOBINclear^TM^ kit (Thermo Fisher Scientific). To minimize batch effects, all samples were processed simultaneously by the same investigator.

RNA samples were submitted for library generation and sequencing by the Beijing Genomics Institute (BGI-Shenzhen). Briefly, poly(A) mRNA was enriched using poly(T) oligo-attached magnetic beads, followed by fragmentation. First strand cDNA synthesis was carried out using random hexamer N6 primers and reverse transcriptase. Following adaptor ligation to cDNA fragments, PCR amplification and purification, single stranded DNA circles were generated in a final library. DNA nanoballs (DNBs) were subsequently generated by rolling circle replication, which underwent paired end sequencing (100 bp) on the BGI DNBseq platform.

### Data Processing and Bioinformatic Analysis

Raw reads were filtered using SOAPnuke (BGI-Shenzhen) and clean reads mapped to the reference human genome (GRCh37) using HISAT2 (Li et al., 2009; Kim et al., 2019). Transcript counts were obtained using RSEM (RNA-Seq by Expectation Maximization) and normalized as FPKM (Fragments Per Kilobase of transcript per Million mapped reads (Li and Dewey, 2011). The DESeq2 algorithm was used to evaluate differentially expressed genes (DEGs) (Love et al., 2014). The *p*-values were adjusted for multiple comparisons by the Benjamini and Hochberg method, and differential expression of the genes was determined using a false discovery rate (FDR) cutoff of <0.05 (Benjamini and Hochberg, 1995). DESeq2 analysis was performed on normalized expression data to include only genes expressed at the level of 1 or higher using default parameters in iDEP. To select DEGs, a fold change of more than ± 2 (log2FC > 1) was used, in view of the relatively low range of dispersion of log2FC values detected.

Principal component analysis (PCA) was used to explore the clustering of data and to characterize the inter-and intra-group variability. Gene expression heatmaps, k-means clustering of DEGs and analysis of tissue-specific co-expression patterns were explored using iDEP.91^1^ and the TopFunn module of the TopGene Suite software (Division of Biomedical Informatics, Cincinnati Children’s Hospital Medical Center, Cincinnati, OH) (Chen et al., 2009; Ge et al., 2018). This analysis relates the observed DEGs’ patterns to specific tissues and cell types.

To gain insight into the functional attributes of DEGs, pathway analysis was carried out using iPathwayGuide (Advaita Bioinformatics, Michigan, United States) and Reactome (Ahsan and Drăghici, 2017; Jassal et al., 2020). iPathwayGuide implements an “Impact Analysis” approach that takes into consideration the direction and type of all signals on a pathway to identify putative mechanisms that can explain the measured gene expression changes (Donato et al., 2013). Pathways were analyzed in the context of data obtained from both Kyoto Encyclopedia of Genes and Genomes (KEGG) and Reactome databases, and ontology data was analyzed using the Gene Ontology Consortium database (Ashburner et al., 2000; Kanehisa and Goto, 2000). Functional analysis was carried out using both iPathwayGuide and Reactome in view of discrepancies in pathway resources between databases that limit interoperability and might influence results (Domingo-Fernández et al., 2018). DEGs were also evaluated by comparison to published datasets describing the acute effects of scuba diving on the peripheral blood transcriptome (Eftedal et al., 2013). Enrichr was used to explore transcriptional regulatory networks and to identify transcription factors (TFs) enriched for target genes using the gene set library from the ChIP-x Enrichment Analysis (ChEA) database (Lachmann et al., 2010; Chen et al., 2013).

### Summary Sequencing Metrics

For all samples, sequencing generated approximately 6 Gbp of data, with Q20 scores over 98% and a median mapping ratio of 72% (IQR 1.5). Using DESeq2, we identified 159 DEGs in the comparison between uneventful diving controls at T1 and DCS cases at T1 (130 upregulated genes in DCS, 29 downregulated genes in DCS)—Supplementary Tables 2, 3. For the comparison of DCS cases at T1 with DCS cases at T2, 300 DEGs were identified (41 upregulated in DCS cases at T2, 259 downregulated in DCS cases at T2)—Supplementary Tables 4, 5. No transcripts exceeded significance thresholds for the comparison between controls at T1 vs. controls at T2. Pairwise comparison of controls at T2 with DCS cases at T2 identified only three transcripts which were significantly upregulated in DCS—Supplementary Table 6.

### qPCR Validation

Independent validation of RNA sequencing data was sought by evaluating the direction and magnitude of differential expression for selected genes using two-step reverse transcription—quantitative PCR (RT-qPCR). Five genes were randomly selected from the DEG pool (*PTGDR2*, *G0S2*, *AREG*, *IL5RA*, and *BMX*). Primers were designed using Primer3 and verified for specificity using NCBI-BLAST (Rozen and Skaletsky, 2000; Kent, 2002). Two hundred nanograms of RNA per subject was used for cDNA library synthesis using the GoScript^TM^ Reverse Transcription System (Promega Corporation). The first-strand cDNA synthesis reaction was primed with oligo(dT) primers. Following reverse transcription, a 1:5 dilution of the cDNA library was used as a template for a quantitative PCR reaction. qPCR was carried out in triplicate using an EvaGreen^®^ Master mix (Solis BioDyne, Estonia) and a BioRad CFX96 instrument. The relative expression of each gene was determined using the 2^–ΔΔCT^ method. Glyceraldehyde-3-phosphate dehydrogenase (*GAPDH*) was used as housekeeping gene reference standard. Primer sequences are listed in the Supplementary Material. Expression patterns of the five selected transcripts matched the direction of expression observed from the RNA-Seq data (Supplementary Material).

### Statistical Analysis

Analysis of clinical data was performed using IBM SPSS v26. The Kolmogorov-Smirnov and Shapiro-Wilk tests as well as visual inspection of Q-Q plots were used to assess the normality of distribution of quantitative variables. Since most variables showed a skewed, non-normal distribution, non-parametric statistics were used. Pairwise comparison between case and control groups was carried out using the Mann-Whitney *U* test for continuous and ordinal dependent variables. Categorical dependent variables were compared using Fisher’s exact test. Numerical data is presented as medians (interquartile range) and categorical data is presented as percentages. A *p-*value of < 0.05 was considered statistically significant.

## Results

### Clinical and Dive Characteristics of the Study Cohort

The baseline clinical characteristics of the seven divers with clinical DCS and six unaffected controls who met the inclusion criteria is shown in Table 1, and detailed dive characteristics in Table 2. No statistically significant differences in baseline demographics, dive characteristics and the Hempleman stress index between case and control subjects were present. No difference in median C-reactive protein (CRP) levels between cases and controls at T1 was detected (0.7 mg/L vs. 1.3 mg/L, *p* = 0.223). Cases at T2 had significantly higher CRP levels than cases at T1 [5.5 (5.45) mg/L vs. 0.7 (1.5) mg/L, *p* = 0.015]. In the DCS cases analyzed, the time from surfacing to onset of symptoms was ≤60 min, with a mean time of 26 min. On first review by the diving medicine physician at the accident and emergency department, all seven cases manifested the typical cutis marmorata rash together with typical history, confirming the diagnosis of DCS. In six cases, this was the first ever episode of DCS. Three cases demonstrated multisystem involvement, and two of these were neurological-type DCS (vestibulocochlear or cerebral). There was no evidence of spinal cord involvement in any of the DCS subjects. One subject presented with limb pain; however, this was not exclusive limb-pain DCS since it was accompanied by a typical cutis marmorata skin rash and preceded by dyspnea. Four cases were cutaneous-only type DCS. Further details according to the Francis and Smith classification for dysbaric illness are provided in Supplementary Table 9. Of note, three cases required further hyperbaric treatments after their initial emergency DCS hyperbaric treatment.

**TABLE 1:** Clinical characteristics of the study group.

**Characteristics**	**DCS cases (*n* = 7)**	**Controls (*n* = 6)**	***p-*value**
**Age**	35 (6)	40 (2)	0.133
**Gender:**			
Males n(%)	5 (71)	4 (67)	
Females n (%)	2 (29)	2 (33)	0.999
Body mass index (kg/m^2^)	26.3 (4.0)	26.5 (1.9)	0.568
**Previous DCS:**			
Yes n (%)	1 (14)	1 (17)	
No n (%)	6 (86)	5 (83)	0.999
**Allergy/inflammatory conditions requiring daily medication:**			
Yes n (%)	1 (14)	2 (33)	
No n (%)	6 (86)	4 (67)	0.559
**Drug history:**			
Yes n (%)	1 (14)	3 (50)	
No n (%)	6 (86)	3 (50)	0.266
**Illicit drug use within the previous 1 month:**			
Yes n (%)	2 (29)	0 (0)	
No n (%)	5 (71)	6 (100)	0.462
**Alcohol within 24 h of the first dive of the day:**			
Yes n (%)	6 (86)	3 (50)	
No n (%)	1 (14)	3 (50)	0.266
**Smoking Status:**			
Current Smoker (%)	3 (43)	2 (33)	
Ex-smoker n (%)	0 (0)	1 (17)	
Non-smoker n (%)	4 (57)	3 (50)	0.999
**Caffeine intake on the day of the dive:**			
Energy drink n (%)	1 (14)	1 (16)	
Coffee n (%)	4 (57)	4 (67)	
Tea n (%)	0 (0)	0 (0)	
Nil n (%)	2 (29)	1 (16)	0.681

*Numerical data is presented as medians with interquartile ranges, categorical data is described as percentages. The interquartile range represents the difference between the 75th and 25th percentiles. Column values are compared by Mann-Whitney *U* test for quantitative variables and by Fisher’s exact test for categorical variables.*

**TABLE 2:** Detailed dive and sampling time characteristics.

**Characteristics**	**Cases (*n* = 7)**	**Controls (*n* = 6)**	***p-*value**
**Gas burden:**			
Low n(%)	3 (43)	3 (50)	
Medium n(%)	4 (57)	3 (50)	
High n(%)	0 (0)	0 (0)	0.999
Hempleman’s stress index (ATA.min^0.5^)	29.9 (6.9)	31.6 (3.7)	0.589
Bottom time (mins)	25.0 (15.5)	21.5 (4.5)	0.385
Total dive time (mins)	55.0 (18.5)	59.5 (13.8)	0.830
Maximum depth (msw)	31.0 (3.8)	32.2 (2.4)	0.315
Time between surfacing and sampling time 1 (h)	2.7 (0.5)	3.8 (1.3)	0.475
Time between surfacing and sampling time 2 (h)	42.9 (2.5)	43.7 (2.6)	0.775

*Quantitative variables are presented as medians with interquartile ranges. The interquartile range represents the difference between the 75th and 25th percentiles. Categorical data is presented as percentages. Column values are compared by Mann-Whitney *U* test for quantitative variables and by Fisher’s exact test for categorical variables.*

### DCS Cases and Diving Controls at T1

The molecular signature of the identified DEGs was interrogated using gene enrichment and pathway perturbation analysis. In the comparison of DCS cases vs. diving controls at T1, statistical enrichment of genes driving cell proliferation in the colorectal cancer and PI3K-AKT signaling pathways was detected. Additionally, significant enrichment for transcripts with immune function in malaria, rheumatoid arthritis, C-type lectin receptor signaling, and Toll-like receptor signaling pathways was observed (Table 3). The top upregulated transcripts in this group include *AREG* (Amphiregulin) and *EREG* (Epiregulin), *FOS* and *G0S2*. *AREG* and *EREG* are secreted peptide autocrine hormones and members of the epidermal growth factor (EGF) family of proteins. They are involved in a wide range of biological processes including inflammation, wound healing, and cell proliferation (Zaiss et al., 2015). The *FOS* gene encodes a leucine zipper protein that dimerizes with proteins of the JUN family, thus forming transcription factor complex AP-1. It regulates cell proliferation, differentiation, and transformation. Expression of the FOS has also been associated with apoptotic cell death and muscle injury (Preston et al., 2000). The phosphatidylinositol 3’-kinase (PI3K)-AKT signaling pathway is activated by many types of cellular stimuli or toxic insults and regulates fundamental cellular functions such as transcription, translation, proliferation, growth, and survival. AKT functions in the control of key cellular processes by phosphorylating substrates involved in apoptosis, protein synthesis, metabolism, and cell cycle. Additionally, early response genes were observed to be upregulated in this comparison.

**TABLE 3:** The top overrepresented pathways identified through impact analysis of DEGs in the casesT1-controlsT1 comparison.

**Pathway**	**Pathway ID**	**No. of genes (DE/All)**	***p*-value**	**Overrepresented genes on pathway**
Colorectal cancer	KEGG–05210	3/85	2.593 e-4	*AREG*, *EREG*, *FOS*
PI3K-AKT signaling	KEGG–04151 Reactome–R-HSA-2219530	8/314	2.617 e-4	*AREG*, *THBS1*, *EREG*, *KITLG*, *ANGPT1*, *SGK2*, *TLR4*, *GNG2*
Malaria	KEGG–05144	4/48	0.002	*THBS1*, *CXCL8*, *SDC2*, *TLR4*
Rheumatoid arthritis	KEGG–05323	5/85	0.003	*CXCL8*, *ANGPT1*, *CCL3*, *TLR4*, *FOS*
C-type lectin receptor signaling	KEGG–0425	4/103	0.003	*EGR3*, *CLEC4D*, *CLEC4E*, *CLEC6A*
Toll-like receptor signaling	KEGG–04620	5/93	0.006	*CXCL8*, *CCL4*, *CCL3*, *TLR4*, *FOS*
Cytokine signaling in the immune system	Reactome–R-HSA-1280215	9/676	0.0155	*FOS*, *IRS2*, *IL1R2*, *MAPK8*, *LGALS9*, *BCL2*, *SERPINB2*, *CCL4*

**FIGURE 1:**
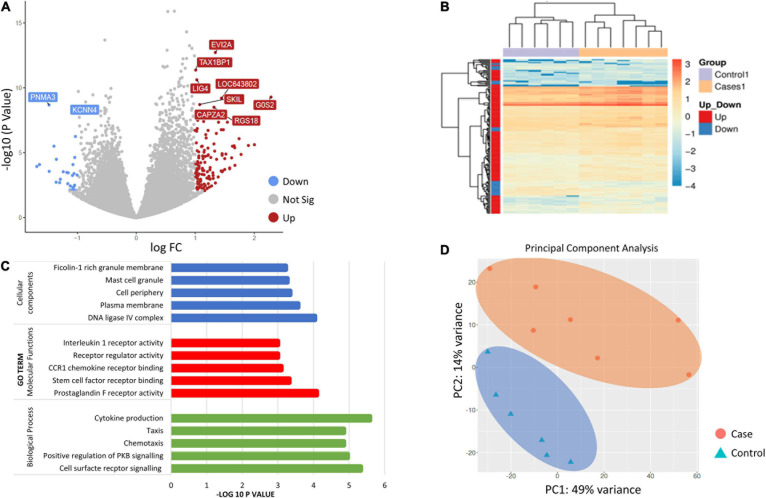
**(A)** Volcano plot showing the top 10 significant differentially expressed genes (DEGs between controls and DCS cases at T1. The volcano plots distribution of log fold change (x-axis) and the negative log (base 10) of the *p*-values (y-axis). Upregulated genes are shown in of red while downregulated genes are shown in blue. **(B)** Heatmap of DEGs for the comparison of controls and cases at T1. Genes on heatmap are organized by hierarchical clustering based on the overall similarity in expression patterns. **(C)** Bar plots showing the top five enriched Gene Ontology terms. **(D)** Principal component analysis (PCA) depicting sample relationship based on dynamic gene expression. PCA identified two clusters in the data separated along the first and second principal components. The percentages on each axis represent the percentages of variation explained by the principal components. PC1 and PC2 define 49 and 14% of the variance, respectively. The distance between the points reflects the variance in gene expression between them. No significant overlap in datapoints representing cases and controls occurred, indicating relative uniformity in gene expression signatures between cases and controls.

Gene ontology (GO) category enrichment analysis showed statistical overrepresentation of transcripts involved in chemotaxis, cell surface receptor signaling and cytokine production (GO—Biological processes). PCA revealed a difference in transcriptome profile between DCS cases and controls at T1, with the first two principal components explaining 63% of the variability across the two biological groups. A greater heterogeneity in transcriptomic profiles amongst replicates in the DCS group was observed, which can potentially be attributed to clinical heterogeneity. No significant overlap in datapoints representing cases and controls occurs, indicating relative uniformity in gene expression signatures within cases and control groups (Figure 1).

### Comparison DCS Cases at T1 and T2

**FIGURE 2:**
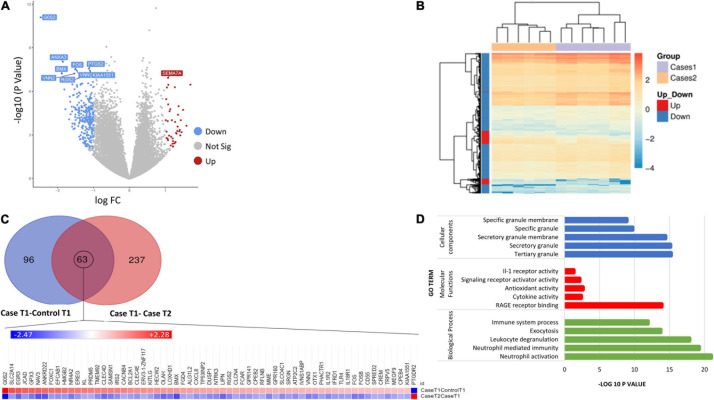
**(A)** Volcano plot showing the top 10 significant DEGs between DCS cases at T1 and DCS at T2. Upregulated genes are shown in red while downregulated genes are shown in blue. **(B)** Heatmap of differentially expressed genes (DEGs) for the comparison of DCS cases at T1 vs. DCS cases at T2. Genes on heatmaps are organized by hierarchical clustering based on the overall similarity in expression patterns. The volcano plots distribution of log fold change (x-axis) and the negative log (base 10) of the *p*-values (y-axis). **(C)** Venn diagram illustrating DEG overlap between the caseT1-controlT1 and caseT2-controlT2 comparison. 63 transcripts at the intersection showed significance in differential expression across both comparisons. The heat map shows that the direction of expression of these transcripts is reversed at T2, suggesting that the resolution of DCS or the exposure to hyperbaric oxygen impacts on their expression. **(D)** Bar plots showing the top 5 enriched Gene Ontology terms for the caseT2-caseT1 comparison.

The comparison of gene expression signatures between DCS cases at T1 and T2 revealed significant differences in several transcripts (Figure 2). Pathway analysis of the DEGs exceeding significance thresholds for the casesT1-casesT2 comparison revealed statistical enrichment of the neutrophil degranulation, signaling by interleukins, IL-10 signaling, IL-4 and IL-13 signaling, and immune system pathways (Table 4). Importantly, almost all transcripts in these pathways were observed to undergo significant downregulation at T2. Gene ontology category analysis revealed overrepresentation of transcripts involved in neutrophil activation and degranulation (GO-biological process), RAGE receptor binding (GO-molecular function), and secretory granules (GO-cellular components).

**TABLE 4:** The top overrepresented pathways identified through impact analysis of DEGs in the casesT1-casesT2 comparison.

**Pathway**	**Pathway ID**	**No. of genes (DE/All)**	***p*-value**	**Overrepresented genes on pathway**
Neutrophil degranulation	R-HSA-6798695	43/480	4.44 e-16	*CDA*, *ORM1*, *TNFAIP6*, *HP*, *SLC2A3*, *CXCL1*, *GPR84*
Signaling by interleukins	R-HSA-449147	33/639	9.88 e-7	*CXCL8*, *CEBPD*, *IL1R1*, *IL1R2*, *IL34*, *ALOX15*, *OSM*, *IL5RA*, *CXCL1*, *IRS2*, *FOS*
Il-4 and Il-13 signaling	R-HSA-6785807	16/211	8.33 e-6	*SOCS3*, *IL1A*, *CXCL8*, *CEBPD*, *BCL6*, *ALOX15*, *OSM*, *FOS*, *PTGS2*, *MMP9*
Immune system	R-HSA-168256	87/2,869	3.23 e-5	*CDA*, *ORM1*, *CXCL8*, *TNFAIP6*, *HP*, *IL5RA*, *SLC2A3*, *CXCL1*, *IRS2*, *PYGL*
Il-10 signaling	R-HSA-6783783	9/86	7.75 e-5	*IL1A*, *CXCL8*, *IL1R1*, *IL1R2*, *CXCL1*, *FPR2*, *PTGS2*
TNF signaling	KEGG–4668	7/110	0.0006	*MMP9*, *SOCS3*, *FOS*, *PTGS2*, *CXCL1*, *CREB6*, *IL18R1*

To better characterize the time course of events following the initial DCS event, we evaluated similarities and differences with the DEGs arising from the case-control at T1 analysis. Ninety-six genes were exclusive to the comparison between casesT1-controlsT1, 237 genes were exclusive to the comparison between casesT1 and casesT2, and 63 genes were in common to both datasets, i.e., they were identified to be differentially expressed in both comparisons (Figure 2). Mechanistically, it is plausible to propose that the 96 genes which were exclusive to the casesT1-controlsT1 comparison represent the early phase of the response to DCS. Furthermore, the 237 genes which were exclusive to the casesT1-casesT2 comparison represent a combination of (a) the later (delayed) manifestations of DCS, (b) the pathophysiological response to DCS involving repair and recovery mechanisms, and/or (c) the physiological response to HBO. All DCS cases recruited in this study made complete clinical recovery by T2, hence it is unlikely that the 237 transcripts unique to the casesT1-casesT2 comparison reflect an ongoing or sustained response to DCS. Nevertheless, a prolonged subclinical pathophysiological reaction cannot be excluded.

To further relate the transcriptomic signature to the clinical resolution of DCS and the effects of HBO, we evaluated the direction of gene expression change in the DEG list at the intersection of the two datasets. These 63 transcripts exceeded statistical thresholds of significance in both the casesT1-controlsT1 and casesT1-casesT2 comparison, suggesting that their expression is causally related to the development and/or progression of DCS. Interestingly, for all the 63 genes, the direction of expression was completely reversed in cases at time point 2 compared to cases at time point 1, indicating that the resolution of DCS and the exposure to HBO significantly impacts on their expression. Pathway enrichment analysis applied to the 63 DEGs at the intersection of the two comparison reveals statistical overrepresentation of PI3K signaling (*p* = 4.8 × 10^–3^), consistent with earlier results demonstrating significant perturbation of the PI3K-AKT pathway in DCS cases compared to controls.

**FIGURE 3:**
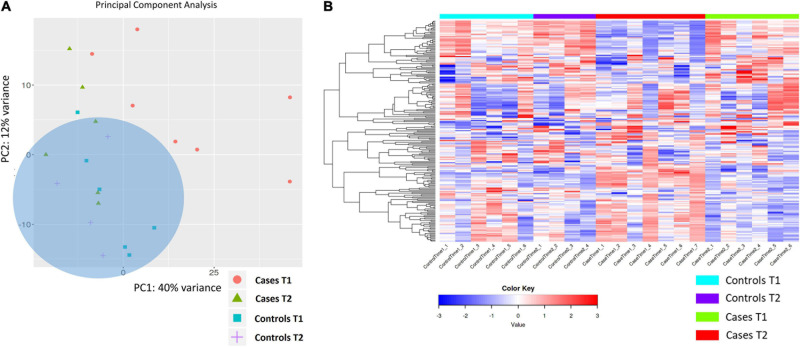
**(A)** PCA analysis of gene expression across sequenced libraries repeated for DCS cases at T1 and T2 and uneventful diving controls at T1 and T2. PC1 and PC2 define 40 and 12% of the observed variance. A third component, PC3, defines 8% of the variance (not shown). Importantly, two distinct but broad clusters can be detected, largely defined by DCS cases at T1 and most of the controls in aggregate. **(B)** Heat map showing the top 200 DEGs across the four biological groups in this study. Genes on heatmap are organized by hierarchical clustering based on the overall similarity in expression patterns. Upregulated genes are shown in shades of red while downregulated genes are shown in shades of blue.

Exploratory PCA analysis was also applied to gene expression data from the four biological groups (DCS cases at T1 and T2, uneventful diving controls at T1 and T2 considered in aggregate). PCA shows absence of separation between DCS cases at T2 and uneventful diving controls, while DCS cases at T1 demonstrated within-group heterogeneity and clustered differently from controls. Two distinct but broad clusters can be detected, largely defined by DCS cases at T1 and most of the controls (Figure 3). This analysis demonstrates that divers after uneventful diving demonstrate a relatively distinct gene expression signature from DCS cases. A greater heterogeneity in transcriptomic response can be observed in divers with DCS at times 1 and 2. It is plausible to propose that this heterogeneity in part reflects the combined effects of clinical treatment and varying resolution of the inflammatory stimulus.

### K-Means Clustering and Analysis of Tissue-Specific Coexpression Patterns Reveals Leukocyte Subtype-Specific Gene Expression Signatures

**FIGURE 4:**
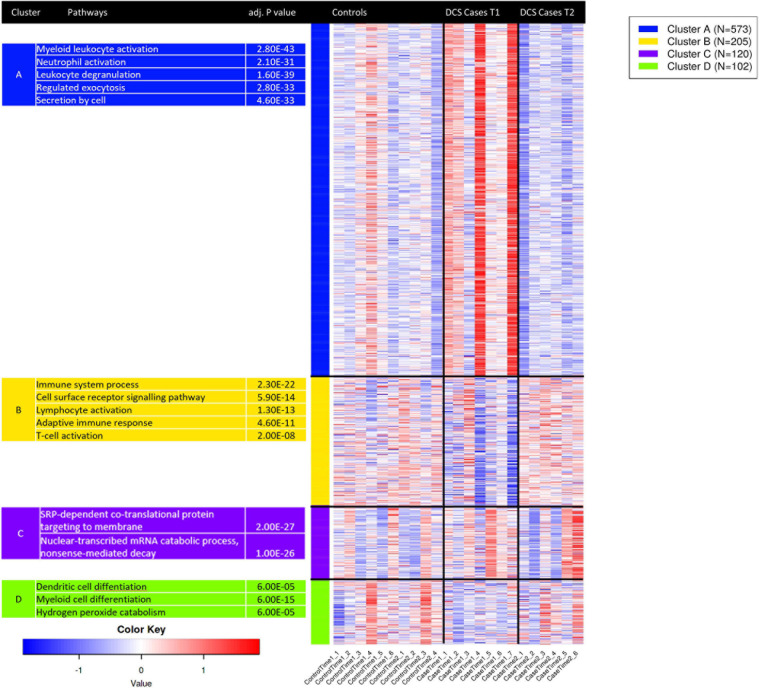
K-means clustering analysis applied to the top 1,000 variable genes identifies co-regulated and co-functional transcripts. Genes in cluster A, strongly enriched for neutrophil activation, degranulation and myeloid activation are variably upregulated in DCS cases at T1. Genes in cluster B, strongly enriched for lymphocyte activation and T—cell activation are downregulated in DCS cases at T1. As GO terms are related or highly redundant, for each cluster the most significant terms are presented. Upregulated genes are shown in shades of red while downregulated genes are shown in shades of blue.

K-means clustering was used to group gene expression signatures and to explore the GO terms enriched for each cluster. This unsupervised method enables the identification of transcripts that are co-functional and are coregulated. Based on the within-group sum of squares, *k* = 4 was chosen. The top 1,000 variable genes were included the analysis. K-means cluster analysis shows that genes in cluster A, strongly enriched for myeloid activation, neutrophil activation and leukocyte degranulation are upregulated in DCS cases at T1, while genes in cluster B, enriched for immune system processes, cell surface receptor signaling and lymphocyte activation were upregulated at T2 in DCS cases. Genes in clusters A and B showed opposing pattern of expression between DCS cases at T1 and T2, with expression patterns at T2 in DCS cases being largely similar to that observed in controls (Figure 4). To further relate the shift in gene expression patterns with specific leukocyte subtypes, we performed an analysis of tissue-specific patterns using ToppFun. The upregulated gene set in DCS cases at T1 are characteristic of myeloid cells, specifically CD11b + Ly6-G + neutrophils, monocytes, macrophages, and some dendritic cell subtypes (19/409 genes in annotation, *p* = 2.029 × 10^–12^). These cells produce Type I interferons and large quantities of reactive oxygen species (ROS). In keeping with K-means clustering analysis, the downregulated genes in DCS cases at T2 are again characteristic of myeloid cells.

### Transcriptional Regulatory Networks

Transcriptional regulatory networks were explored using enrichment analysis of TF target gene sets. For the case T1-control T1 comparison, target genes of *SP1* (FDR = 5.9 × 10^–2^), *GLIS2* (FDR = 1.1 × 10^–2^) and *PAX5* (FDR = 1.5 × 10^–1^) were overrepresented in down-regulated genes, while upregulated genes are enriched with target genes of *HLF* (FDR = 8.6 × 10^–2^) and *FOXK1* (FDR = 1.3 × 10^–1^). For the case T2-caseT1 comparison, target genes of *CEBPB* (FDR = 4.82 × 10^–4^) and *SCL* (FDR = 1.73 × 10^–3^) were overrepresented in upregulated genes, while downregulated genes were enriched for targets of *BACH1* (FDR = 2.984 × 10^–7^) and *ZNF217* (FDR = 3.2 × 10^–5^). *SP1*, *GLIS2*, *SCL*, and *FOXK1* play pivotal roles in cycle regulation. *PAX5* is a known regulator of B-cell development and *CEBPB* regulates the expression of genes involved in acute phase immune and inflammatory responses (Cobaleda et al., 2007, p. 5; Wang W. et al., 2015).

## Discussion

This study describes changes in the peripheral blood leukocyte transcriptome in divers with cutaneous DCS compared to matched controls after uneventful diving. It also evaluates the progression of these transcriptomic changes by comparison of gene expression at two discrete timepoints. We show that cutaneous DCS elicits the differential expression of several transcripts involved in leukocyte activity, inflammation, and cytokine production, with prominent perturbation of genes in the PI3K-AKT and TLR pathways. In fresh DCS cases within 8 h of surfacing from diving, upregulated transcripts are characteristic of the leukocyte myeloid lineage—specifically granulocytes. These show a significant downregulation when evaluated just under 48 h later. The reversal in the direction of expression of these transcripts is likely attributed to the effects of HBO and the clinical resolution of DCS. To the best of our knowledge, this is the first study that evaluates DCS-induced transcriptomic alterations in man.

### DCS Triggers Activation of PI3K-Akt, TLR Pathways, and Immediate Early Genes

The findings from this study require meaningful interpretation in the context of existing knowledge on DCS. DCS is a systemic pathological process arising from the formation of inert gas bubbles in supersaturated tissues or blood following decreases in ambient pressure (Vann et al., 2011). Damage and dysfunction of the vascular endothelium following decompression has been reported in a number of animal studies (Nossum et al., 1999, 2002). Decompression-induced endothelial dysfunction correlates with bubble formation in rat models of DCS, an effect which is potentially attenuated by immunomodulatory drugs such as simvastatin or by NO donors (Møllerløkken et al., 2006; Zhang et al., 2015, 2016). In our study, acute phase DCS was accompanied by upregulation of genes involved in acute inflammation (*AREG*, *EREG*, *CXCL8*, *CCL4*, *EGR3*, *CLEC4E*, *CLEC6A*) and innate immunity (*TLR4*, *EGR3)*. Amphiregulin (*AREG*) and epiregulin (*EREG*) are type II cytokines linked to a wide variety of inflammatory conditions, such as rheumatoid arthritis, chronic airway disease and glomerulonephritis (Yamane et al., 2008; Melderis et al., 2020). *AREG* skews monocytes and macrophages into a proinflammatory M1 phenotype, and the expression of *EREG* is induced by intermittent hypoxia (Kyotani et al., 2018). Toll-like receptors function in activation of the innate immune system, and *CXCL8* was the most strongly upregulated DEG for this pathway. *CXCL8* (IL-8) is a powerful chemoattractant involved in neutrophil recruitment and chemotaxis. *CXCL8* and other chemokines are consistently among the first signals to be expressed and released by the various cell types involved in inflammation (de Oliveira et al., 2013; Torraca et al., 2017). Animal studies have demonstrated that acute DCS is accompanied by elevation of proinflammatory cytokines (TNF-α, IL-6, and IL-1) in both lung tissue and the circulation (Bigley et al., 2008; Chen et al., 2011). Thus, the proinflammatory gene expression signature of acute DCS described here is in keeping with evidence from previous *in vivo* animal studies. The acute-phase gene expression signature described in this report is significantly enriched for neutrophil and monocyte/macrophage-specific genes. Critically, this pattern is reversed at 40–44 h after surfacing from diving, following exposure to HBO. The role of neutrophil activation and vascular damage following microparticle release has been well-documented, with studies showing that exposure to inert gases at high pressure generates oxidative stress (Thom et al., 2011, 2014, 2015). Furthermore, uneventful diving triggers a similar shift in transcriptional pattern, with upregulation of genes expressed by myeloid cells and down regulation of CD8+ lymphocyte-expressed genes (Eftedal et al., 2013). Importantly, Eftedal et al. (2013) did not assess DCS or HBO exposures. The gene expression patterns described here are capturing the combined effect of three interrelated physiological stressors—diving, DCS and HBO exposure. At the two timepoints analyzed, these factors exert a varying effect on the magnitude and direction of the observed gene expression changes. It is likely that the diving-induced transcriptomic changes in the pathways observed by Eftedal et al. (2013) may have been obscured or possibly reversed in this study cohort. Furthermore, it is plausible to postulate that a direct continuum exists between the changes induced by uneventful diving and development of DCS, with the latter developing if the adverse physiological stimulus is in any way prolonged or sustained. Uneventful diving triggers a subclinical inflammatory response that can possibly progress to clinically overt DCS in susceptible individuals under extreme physiological conditions. In such a scenario, it is possible to hypothesize that the recruitment and activation of additional inflammatory pathways would potentially account for the observed clinical picture of DCS.

Pathway analysis in acute DCS demonstrates significant overrepresentation of PI3K-AKT and Toll-like receptor (TLR) signaling. The PI3K-AKT pathway has diverse cellular roles as it regulates proliferation, metabolism, survival, migration and tumor initiation (Cantley, 2002; Manning and Cantley, 2007). AKT positively regulates the production of reactive oxygen/nitrogen species (ROS/RNS) by modulating mitochondrial bioenergetics and activation of NADPH oxidases (Koundouros and Poulogiannis, 2018). TLR2 and TLR4 recognize pathogen-associated molecular patterns (PAMPs) on bacterial cell walls and activate innate immunity through NF-κB activity. TLR signaling augments activity of the PI3K-AKT pathway, and TLR2 or TLR4 activation on myeloid cells promotes the production of pro and anti-inflammatory cytokines (Weichhart et al., 2015). Recently, HBO therapy was shown to reverse AKT activity in a rat model of neuropathic pain (Liu et al., 2019). Importantly, Chen et al. (2011) showed that exposure to compression-decompression activates AKT signal transduction in the rat lung.

In addition, several other DEGs identified from the comparison of cases and controls within 8 h of surfacing from a dive are physiologically important. Of note, *EGR3* (Early Growth Response 3) was upregulated in DCS cases at T1. This transcription factor induces TGF-β, is a negative regulator of T-cell activation and is necessary for humoral immune tolerance (Morita et al., 2016). *JCAD* (Junctional Cadherin 5 Associated) is a junctional protein linked to proinflammatory changes and dysfunction of vascular endothelium (Shigeoka et al., 2020). *THBS1* (Thrombospondin 1) is a glycoprotein secreted by endothelium, where it participates in a broad array of functions related to vascular inflammation and cell-cell interactions (Liu Z. et al., 2015). *NR4A2* (Nuclear Receptor Subfamily 4 Group A Member 2) encodes an orphan nuclear receptor belonging to the steroid-thyroid hormone-retinoid receptor superfamily. *NR4A2* has also been shown to mediate acute inflammatory cascades (Doi et al., 2008). Members of the *NR4A* family are classified as “immediate early genes” (IEGs) induced by physiological and physical stimuli (Maxwell and Muscat, 2006). IEGs constitute a gateway to genomic responses, being rapidly and transiently activated in response to multiple stimuli. Their induction underlies acute inflammation (Wu et al., 2019). Gene expression studies following simulated diving in rats exposed to high bubble loads showed upregulation of *NR4A3*, a paralog of *NR4A2* (Eftedal et al., 2012). *G0S2* (G0/G1 switch gene 2) was significantly upregulated in DCS cases at T1. *G0S2* has disparate roles and is implicated in hypoxia-induced positive regulation of oxidative phosphorylation and induction of apoptosis by interaction with BCL2. *G0S2* is also an early response gene that is upregulated in autoimmune and inflammatory processes, and functions in the maintenance of T-cell quiescence (Yamada et al., 2011; Lee et al., 2015).

### DCS Induces Activation of Antioxidant Defense Mechanisms

Our investigation also showed a significant upregulation of *GPX3* (Glutathione Peroxidase 3) in DCS cases at T1. This is an extracellular selenocysteine protein that plays a critical role in the scavenging of ROS. The upregulation of anti-oxidant pathways including *SOD2* and *GPX4* in diving has been described (Eftedal et al., 2013). In our study, *SOD2* was strongly expressed in both DCS cases and controls at T1, with a higher expression in DCS cases that, however, did not reach significance thresholds after adjustment for multiple testing. It is reasonable to postulate that *SOD2* was upregulated in response to diving in both DCS cases and controls, in keeping with previous studies. *SOD2* expression did not differ between controls at T1 and controls at T2. This is consistent with the findings of Perović et al. (2018) where the expression of *SOD2* normalized within 6 h post dive. In the casesT1-casesT2 comparison, nominal but significant downregulation of *SOD2* was observed at T2. Critically, extracellular superoxide activates neutrophils in a TLR4 dependent manner, and sub-lethal oxidative stress leads to selective activation of monocytes/macrophages through scavenger receptors (Lorne et al., 2008; Geiger-Maor et al., 2012).

### Effect of HBO on the Peripheral Blood Transcriptome

The leukocyte gene expression patterns reported here are biologically meaningful. In acute DCS, the activation of inflammatory pathways playing critical roles in body defense is likely reflecting a physiological reaction triggered by a powerful exogenous stressor. However, the effect of HBO on gene expression is controversial and not fully ascertained. The transcriptomic changes in DCS cases at T2 are driven by the combined effects of clinical resolution of DCS and HBO therapy. This study was not designed to examine the relative contribution of each factor, and to the best of our knowledge the impact of HBO on the leukocyte transcriptome in man has not been reported. Limited inferences can be drawn from the literature. Godman et al. (2010) reported that hyperbaric exposure triggers the upregulation of antioxidant, cytoprotective, and IEGs in human microvascular endothelial cells, rendering them resistant to increased oxidative stress. Woo et al. (2020) showed that HBO alleviates exercise-induced inflammatory responses and muscle damage. Other investigators showed that a single HBO treatment significantly alters the expression of different inflammation and wound healing genes in endothelial cells at different timepoints (Kendall et al., 2012). HBO therapy is considered a useful anti-inflammatory adjunct in various pathologies (Novak et al., 2016; Hao et al., 2020). Conversely, HBO did not induce any pro-inflammatory, anti-inflammatory, or antioxidant effects in surgically induced inflammation in dogs (Gautier et al., 2019). Recently, a microarray transcriptome study showed that HBO does not exert significant effects on gene expression during surgical wound healing in rabbits (Tlapák et al., 2020). Critically, direct comparison of these studies is limited by differences in exposure, species, and the analytic methodology applied.

### The DCS Transcriptome—Caveats and Challenges

The interpretation of transcriptomic signatures in DCS is challenging. Primarily, the DCS phenotype is clinically heterogenous, an effect which might account for within-group dispersion of gene expression. Additionally inter-subject and intra-subject variation in peripheral blood is well-recognized due to the dynamic nature of this tissue. In attempt to increase homogeneity in the DCS case cohort, we selected to only include divers exhibiting the cutis marmorata rash. This cutaneous manifestation is indicative of a systemic DCS pathology and an inflammatory response to nitrogen bubbles (Kalentzos, 2010). Cutis marmorata is not a universal feature of DCS and it is possible that the findings reported here are skewed toward one clinical subtype of DCS, and thus may not be generalizable to all forms of DCS. Secondly, acclimation to decompression has been described in rodent models (Montcalm-Smith et al., 2007, 2009). Chen et al. (2011) further show that repeated exposure to compression-decompression stress induces changes in EGR-1 and cytokine expression, although no acclimation-specific changes were detected. The physiological and molecular basis of acclimation are unknown, although reduced bubble load and attenuation of host responses have been suggested to play a role (Huang et al., 2003; Pontier et al., 2009). It is likely that the transcriptomic changes reported here are confounded to varying extents by interindividual differences in acclimation responses.

### Are the Observed Transcriptomic Changes Confounded by Diving?

In view of the low incidence of DCS, this investigation was not designed to include pre-dive samples from DCS cases and controls. Pre-dive transcriptomic changes could thus not be directly investigated or considered as potential confounders. Furthermore, it is established that uneventful scuba diving triggers several acute gene-expression changes in blood sampled within 1 h of diving. Eftedal et al. (2013) characterized DEGs involved in apoptosis, immune responses, and inflammatory pathways that can be persistently deregulated in experienced divers. To increase the robustness and significance of this study, we sought to evaluate the DEG profile from the casesT1-controlsT1 comparison in this study to the dataset from Eftedal et al. (2013) showing acute blood transcriptomic changes after uneventful scuba diving. This analysis identified only seven DEGs at the intersection between the two datasets, of which five were consistently overexpressed in both datasets. This suggests that their expression is potentially modulated by the diving process itself rather than DCS. Conversely, two genes—*GZMB* and *ZFYVE16* showed an opposite direction of expression when the two datasets were compared. Interestingly, the magnitude of expression change was higher in DCS cases than in the Eftedal dataset. Eftedal et al. (2013) identified significant enrichment for the pathway containing *GZMB* (granzyme B) in response to uneventful diving, with *GZMB* and related genes undergoing transient change in expression and returning to predive levels between successive dives. In our dataset, *GZMB* underwent significant upregulation in DCS cases compared to diving controls. *GZMB* did not, however, exceed statistical significance thresholds when DCS cases at T2 were included in the comparison, implicating that upregulation of this gene may have persisted past 40–44 h from surfacing from the dive resulting in DCS. Although it cannot be fully evaluated in this study, its sustained upregulation provides evidence for a prolonged or persistent pro-inflammatory response. Similarly, *ZFYVE16* overlapped between the two datasets but showed an opposite direction of expression. The relevance of this locus to DCS biology is unknown. Comparison with the Eftedal dataset provides support for a minimal, yet relevant overlap between physiological responses elicited by uneventful diving and that of DCS. Furthermore, the absence of transcripts exceeding statistical significance thresholds between controls at T1 and controls at T2 reinforces the transient nature of transcriptomic changes, consistent with previous observations (Eftedal et al., 2013). We conclude that at the two sampling timepoints in this study, any gene expression changes induced by uneventful diving were reversed at T2.

### Strengths and Limitations

Our investigation is strengthened by several factors. A unique human genomic signature of DCS has not been reported previously. Here, a biologically agnostic high-throughput RNA sequencing approach is used. This method carries several advantages over microarray-based analysis, including low background noise and a large dynamic range (Hrdlickova et al., 2017). The RNA sequencing data was further validated using RT-qPCR. The selection of subjects followed stringent inclusion and exclusion criteria. To control for gene expression changes that arise due to diving, a comparably matched cohort of divers after uneventful diving was recruited. To ensure that the observed transcriptomic changes are a faithful representation of the DCS state, care was taken to minimize interindividual differences amongst the divers selected for this study. The peripheral blood transcriptome is known to broadly reflect the combined effects of pharmacotherapy, chronic disease, and environmental exposures such as smoking (Lampe et al., 2004; Liu Y. et al., 2015; Beretta et al., 2020; Ustinova et al., 2020). While these factors are integral components of many human studies, they can cause confounding in gene expression experiments.

This investigation carries a number of limitations. Primarily, the study was not designed to include pre-dive samples, hence the baseline stationary transcriptome could not be compared to the DCS changes at T1 and T2. DCS has a low incidence and is inherently unpredictable in nature, thus limiting prospective follow-up studies (Dardeau et al., 2012). Further investigations using simulated diving in animal models are necessary to clearly elucidate the transcriptomic effects of DCS and their time course. The small number of DCS cases meeting inclusion criteria necessitated the use of convenience sampling as opposed to random sampling, and selection bias cannot be fully excluded. In human subjects, the accurate quantification of decompression stress in uncontrolled environments is difficult. Despite our best efforts to ensure phenotypic homogeneity and comparability of the case and control cohorts, it is likely that uncontrolled factors persist. These may include differences in dive profiles and breathing gas composition that could modulate gene expression. The three-tier Francis and Smith classification and Hempleman’s stress index may not fully capture the dynamic variation in dive characteristics or decompression profiles that could impact on the eventual clinical course. Additionally, we acknowledge that the unavailability of dive computer profile data and information on recent earlier diving is a significant limitation. Furthermore, gene expression data was not correlated to the quantitative determination of serum cytokine levels. A poor correlation between RNA expression and protein levels has been reported, in part due to variations in translational efficiency (Schwanhäusser et al., 2011; Cenik et al., 2015). Future research should also strive to evaluate the immunophenotype of peripheral blood leukocytes in response to DCS using flow cytometry to better define the inflammatory landscape in DCS.

Peripheral blood leukocytes were the primary source of RNA. These cells represent a highly heterogenous cell population, and dilution of biological effects due to the heterogenous cell population possibly accounts for the low range of log2FC (log2FC < 3) values detected in this study. In the targeted gene expression diving study by Chen et al. (2011) identically mild changes in cytokine gene expression were detected in rat lung. Of note, blood transcriptomic studies often apply arbitrary ranges of FC cut-offs (Greiner et al., 2013; Gaye et al., 2017; Dorsey et al., 2019; Ramsay et al., 2019). Furthermore, in many conditions, the magnitude of expression change in the global blood transcriptome may be subtle and generally lower than that observed in tissue-based experiments.

We also acknowledge that this investigation is limited by its small scale. While the findings from this pilot dataset offer insight into the transcriptomic responses to DCS, replication and independent validation in larger cohorts is warranted to enable robust and unbiased interpretation. Replication studies should also consider sampling at alternate time points to better elucidate the dynamics of the stress response elicited by DCS and its resolution. RNA-sequencing is, however, a powerful, precise tool in differential gene expression analysis and data robustness is dependent on the quality of each sample. This study is strengthened by the use of RNA exceeding the quality thresholds acceptable for a high throughput sequencing experiment. Quality control procedures following RNA extraction and bioinformatic analysis of sequencing data did not flag any outlier sample with poor quality issues.

Lastly, the paucity of human studies evaluating the DCS transcriptome limits comparison and interpretation of our findings. While comparison to various animal studies has been drawn, it must be emphasized that notable differences exist between rodents and man in the context of the genomic responses to acute inflammation (Osterburg et al., 2013; Seok et al., 2013). Metabolic differences between murine and human M1 macrophages have been described (Vijayan et al., 2019; Palmieri et al., 2020). Thom et al. (2014) showed that human and murine neutrophils exhibited similar responses to high pressure gases, however, murine cell microparticle production persisted even after decompression.

## Conclusion

In this report we characterize the transcriptomic response elicited by DCS and its sequential progression in response to HBO therapy. This is a pilot investigation that provides a unique insight into the molecular etiology of this complex condition. DCS is accompanied by the dynamic regulation of several inflammatory and innate immune pathways, with a pronounced shift in transcriptional patterns characteristic of the myeloid lineage. Activation of free radical scavenging mechanisms is prominent in DCS. Our findings reinforce the role of acute inflammation in DCS and provide evidence for a continuum between the physiological response elicited by uneventful diving and diving complicated by DCS. Future studies with DCS cohorts are required to investigate the immunophenotype of peripheral blood leukocytes, as well as to enquire the epigenetic, metabolomics, and proteomic landscape of this disease.

## Data Availability Statement

The datasets presented in this study can be found in online repositories. The names of the repository/repositories and accession number(s) can be found below: EMBL-EBI (www.ebi.ac.uk/arrayexpress) under accession number E-MTAB-10388. Intermediate analysis files have been uploaded on Dryad repository (doi: 10.5061/dryad.280gb5mpw).

## Ethics Statement

The studies involving human participants were reviewed and approved by the Faculty Research Ethics Committee, University of Malta (FRECMDS_1718_058). The patients/participants provided their written informed consent to participate in this study.

## Author Contributions

KM, IE, LM, CA, SM, and NP: study conception and design. KM and VP: sample and data acquisition. KM, NP, IE, and VP: data analysis and drafting of the manuscript. IE and NP: project supervision. All authors contributed to the article and approved the submitted version.

## Conflict of Interest

The authors declare that the research was conducted in the absence of any commercial or financial relationships that could be construed as a potential conflict of interest.

## Footnotes

1 http://bioinformatics.sdstate.edu/idep/
