# Supplementary material for: Acute Effects on the Human Peripheral Blood Transcriptome of Decompression Sickness Secondary to Scuba Diving
Source: Front Physiol. 2021 Jun 10;12:660402. doi: 10.3389/fphys.2021.660402 (PMC8222921; doi:10.3389/fphys.2021.660402)
Supplement: Supplementary file 1 [file Data_Sheet_1.docx]

Supplementary Material

**Supplementary table 1**

|  | Cases | Controls |
| --- | --- | --- |
| Inclusion Criteria* | Cutis marmorata rash within 8 hours of surfacing from a scuba dive, witnessed and confirmed by diving medicine physician. | Surfaced from a scuba dive with a ≥25 msw maximum depth without dive table violation. |
|  | Presentation at the emergency department within 8 hours of surfacing from a dive. | Surfaced from a dive within the preceding 8 hours. |
|  |  | Subjected to diving physician questioning and physical examination at T1 and T2 to exclude DCS. |
|  | Fasted for 10hrs prior to T2. | Fasted for 10hrs prior to T2. |
|  | Age ≥ 18 years old. | Age ≥ 18 years old. |
| Exclusion Criteria | Refusal of appropriate hyperbaric treatment. | Underwent further diving before T2. |
|  | Performed strenuous exercise between the dive and T2. | Performed strenuous exercise between the dive and T2. |
|  | Ingested alcohol between the dive and T2. | Ingested alcohol between the dive and T2. |
|  |  | Symptoms suggestive of DCS at T1, T2 or between T1 and T2. |
| *All inclusion criteria were required to be satisfied for recruitment. | | |

**Detailed inclusion and exclusion criteria for cases and controls. DCS: decompression sickness, msw: metre sea water.**

**Supplementary table 2**

| **Rank** | **Gene Symbol** | **Official Full Name** | **log_2_FC**  **(Cases1/Controls1)** | ***p*-adjusted** |
| --- | --- | --- | --- | --- |
| 1 | *G0S2* | G0/G1 switch 2 | 2.28 | <0.001 |
| 2 | *AREG* | Amphiregulin | 2.00 | <0.001 |
| 3 | *PTGFR* | Prostaglandin F receptor | 1.90 | <0.001 |
| 4 | *SLC2A14* | Solute carrier family 2 member 14 | 1.78 | <0.001 |
| 5 | *EGR3* | Early growth response 3 | 1.74 | <0.001 |
| 6 | *JCAD* | Junctional cadherin 5 associated | 1.67 | 0.002 |
| 7 | *GPX3* | Glutathione peroxidase 3 | 1.67 | <0.001 |
| 8 | *NAV3* | Neuron navigator 3 | 1.66 | <0.001 |
| 9 | *THBS1* | Thrombospondin 1 | 1.63 | <0.001 |
| 10 | *ANKRD22* | Ankyrin repeat domain 22 | 1.62 | <0.001 |
| 11 | *FOXC1* | Forkhead box C1 | 1.58 | 0.003 |
| 12 | *EFCAB1* | EF-hand calcium binding domain 1 | 1.58 | 0.003 |
| 13 | *HMGB2* | High mobility group box 2 | 1.55 | <0.001 |
| 14 | *NR4A2* | Nuclear receptor subfamily 4 group A member 2 | 1.52 | <0.001 |
| 15 | *EREG* | Epiregulin | 1.50 | <0.001 |

**The 15 most strongly up-regulated genes for the comparison of controls at sampling time 1 with cases at sampling time 1 (sampling of whole blood within 8 hours of surfacing from a compressed gas underwater dive). The positive log_2_FoldChange (log_2_FC) values indicate that genes were up-regulated in cases at sampling time 1: Log_2_FC = Log_2_(Cases1 Expression) − Log_2_(Controls1 Expression).**

**Supplementary table 3**

| **Rank** | **Gene Symbol** | **Official Full Name** | **log_2_FC**  **(Cases1/Controls1)** | ***p*-adjusted** |
| --- | --- | --- | --- | --- |
| 1 | *C4BPA* | Complement component 4 binding protein alpha | -1.68 | 0.002 |
| 2 | *MAPK8IP1* | Mitogen-activated protein kinase 8 interacting protein 1 | -1.64 | 0.002 |
| 3 | *PNMA3* | PNMA family member 3 | -1.48 | <0.001 |
| 4 | *MYOM2* | Myomesin 2 | -1.48 | 0.004 |
| 5 | *CYB5R2* | Cytochrome b5 reductase 2 | -1.39 | <0.001 |
| 6 | *EBF3* | EBF transcription factor 3 | -1.36 | 0.02 |
| 7 | *SLC7A8* | Solute carrier family 7 member 8 | -1.35 | <0.001 |
| 8 | *CHGB* | Chromogranin B | -1.30 | 0.02 |
| 9 | *DSCAML1* | DS cell adhesion molecule like 1 | -1.29 | 0.01 |
| 10 | *TMPRSS9* | Transmembrane serine protease 9 | -1.24 | 0.004 |
| 11 | *RASA4B* | RAS p21 protein activator 4B | -1.18 | 0.005 |
| 12 | *NRG1* | Neuroregulin 1 | -1.16 | 0.02 |
| 13 | *IGSF22* | Immunoglobulin superfamily member 22 | -1.15 | 0.005 |
| 14 | *SLC12A1* | Solute carrier family 12 member 1 | -1.12 | 0.046 |
| 15 | *DOC2A* | Double C2 domain alpha | -1.12 | 0.03 |

**The 15 most strongly down-regulated genes for the comparison of controls at sampling time 1 with cases at sampling time 1 (sampling of whole blood within 8 hours of surfacing from a compressed gas underwater dive). The negative log_2_FoldChange (log_2_FC) values indicate that genes were down-regulated in cases at sampling time 1: Log_2_FC = Log_2_(Cases1 Expression) − Log_2_(Controls1 Expression).**

**Supplementary table 4**

| **Rank** | **Gene Symbol** | **Official Full Name** | **log_2_FC**  **(Cases2/Cases1)** | ***p*-adjusted** |
| --- | --- | --- | --- | --- |
| 1 | *PTGDR2* | Prostaglandin D2 receptor 2 | 1.70 | <0.001 |
| 2 | *PRSS33* | Serine protease 33 | 1.59 | 0.004 |
| 3 | *IL5RA* | Interleukin 5 receptor subunit alpha | 1.50 | 0.001 |
| 4 | *MEIS2* | Meis homeobox2 | 1.46 | 0.008 |
| 5 | *OLIG2* | Oligodendrocyte transcription factor 2 | 1.44 | 0.003 |
| 6 | *LDB3* | LIM domain binding 3 | 1.42 | 0.01 |
| 7 | *UGT2A3* | UDP glucuronosyltransferase family 2 member A3 | 1.40 | 0.007 |
| 8 | *UMODL1* | Uromodulin like 1 | 1.39 | 0.008 |
| 9 | *ADAMTS7* | ADAM metallopeptidase with thrombospondin type 1 motif 7 | 1.38 | <0.001 |
| 10 | *SMPD3* | Sphingomyelin phosphodiesterase 3 | 1.35 | 0.002 |
| 11 | *UGT2B11* | UDP glucuronosyltransferase family 2 member B11 | 1.34 | 0.005 |
| 12 | *PRSS3* | Serine protease 3 | 1.34 | 0.008 |
| 13 | *FAM46B* | Terminal nucleotidyltransferase 5B | 1.32 | 0.01 |
| 14 | *IL34* | Interleukin 34 | 1.30 | 0.009 |
| 15 | *ALOX15* | Arachidonate 15-lipoxygenase | 1.29 | 0.02 |

**The 15 most strongly up-regulated genes for the comparison of cases at sampling time 1 with cases at sampling time 2. The positive log_2_FoldChange (log_2_FC) values indicate that genes were up-regulated in cases at sampling time 2: Log_2_FC = Log_2_(Cases2 Expression) − Log_2_(Cases1 Expression). Sampling time 1 represents collection of whole blood within 8 hours of surfacing from a dive. Sampling time 2 represents sampling at 40-44hrs after surfacing from a dive.**

**Supplementary table 5**

| **Rank** | **Gene Symbol** | **Official Full Name** | **log_2_FC**  **(Cases2/Cases1)** | ***p*-adjusted** |
| --- | --- | --- | --- | --- |
| 1 | *G0S2* | G0/G1 switch 2 | -2.47 | <0.001 |
| 2 | *BMX* | BMX non-receptor tyrosine kinase | -1.88 | <0.001 |
| 3 | *ANXA3* | Annexin A3 | -1.85 | <0.001 |
| 4 | *NAV3* | Neuron navigator 3 | -1.81 | <0.001 |
| 5 | *ANKRD22* | Ankyrin repeat domain 22 | -1.78 | <0.001 |
| 6 | *CRISP2* | Cysteine rich secretory protein 2 | -1.68 | 0.003 |
| 7 | *EGR3* | Early growth response 3 | -1.66 | <0.001 |
| 8 | *LIPN* | Lipase family member N | -1.65 | <0.001 |
| 9 | *OLAH* | Oleoyl-ACP hydrolase | -1.64 | 0.002 |
| 10 | *TNFAIP6* | TNF alpha induced protein 6 | -1.61 | <0.001 |
| 11 | *CLEC4D* | C-type lectin domain family 4 member D | -1.59 | 0.001 |
| 12 | *FABP4* | Fatty acid binding protein 4 | -1.59 | 0.005 |
| 13 | *SLC37A3* | Solute carrier family 37 member 3 | -1.59 | <0.001 |
| 14 | *MMP9* | Matrix metallopeptidase 9 | -1.59 | 0.004 |
| 15 | *ACSL1* | Acyl-CoA synthetase long chain family member 1 | -1.57 | <0.001 |

**The 15 most strongly down-regulated genes for the comparison of cases at sampling time 1 with cases at sampling time 2. The negative log_2_FoldChange (log_2_FC) values indicate that genes were down-regulated in cases at sampling time 2: Log_2_FC = Log_2_(Cases2 Expression) − Log_2_(Cases1 Expression). Sampling time 1 represents collection of whole blood within 8 hours of surfacing from a dive. Sampling time 2 represents sampling at 40-44hrs after surfacing from a dive**

**Supplementary table 6**

| **Rank** | **Gene Symbol** | **Gene Full Name** | **log_2_FC**  **(Cases2/Controls2)** | ***p*-adjusted** |
| --- | --- | --- | --- | --- |
| 1 | *SLC2A14* | Solute carrier family 2 member 14 | 1.69 | 0.0249 |
| 2 | *CCDC163* | Coiled-coil domain containing 163 | 1.5 | 0.0126 |
| 3 | *HOXB2* | Homeobox B2 | 1.05 | 0.0126 |

**The three DEGs which met statistical significance for the comparison of controls at sampling time 2 with cases at sampling time 2 (whole blood sampling at 40-44hrs after surfacing from a compressed gas underwater dive). The positive log_2_FoldChange (log_2_FC) values indicate that genes were up-regulated in cases at sampling time 2: Log_2_FC = Log_2_(Cases2 Expression) − Log_2_(Controls2 Expression).**

**Supplementary table 7**

| **Primer Description** | **Oligonucleotide Sequence** |
| --- | --- |
| *PTGDR2* forward primer | TGG AGT CAT CCT CTT CGT GGT G |
| *PTGDR2* reverse primer | AGT AGG TGA AGA AGG GCA GGG A |
| *IL5RA* forward primer | TGACTGGCTTGCGGTGCTTGTT |
| *IL5RA* reverse primer | CTGCTGTGACATTCAGTGGAGG |
| *GOS2* forward primer | GCCTGATGGAGACTGTGTGCAG |
| *GOS2* reverse primer | TCCTGCTGCTTGCCTTTCTCCT |
| *BMX* forward primer | ACCTGAGGAGTCACGGAAAAGG |
| *BMX* reverse primer | CTGTCCACCAAGCAGTTACGAG |
| *AREG* forward primer | GCA CCT GGA AGC AGT AAC ATG C |
| *AREG* reverse primer | GGC AGC TAT GGC TGC TAA TGC A |
| *GAPDH* forward primer | GTC TCC TCT GAC TTC AAC AGC G |
| *GAPDH* reverse primer | ACC ACC CTG TTG CTG TAG CCA A |

**Oligonucleotide sequences of primers used during qPCR: *PTGDR2* (prostaglandin D2 receptor 2), *IL5RA* (interleukin-5 receptor subunit alpha), *G0S2* (G_0_/G_1_ switch 2), *BMX* (BMX non-receptor tyrosine kinase), AREG (amphiregulin) and *GAPDH* (glyceraldehyde-3-phosphate dehydrogenase, housekeeping gene).**

**Supplementary qPCR data**

The qPCR reaction mixture was composed of 5x HOT FIREPol® EvaGreen® qPCR Mix Plus ROX (12.5mM MgCl_2_, dNTPs (deoxynucleoside triphosphate), EvaGreen® dye and ROX dye – Solis BioDyne, Estonia) together with 10pmoles/µL of each of the forward and reverse primers and molecular biology grade water. Reactions were carried out in a final volume of 20µL, using 1µL of the diluted cDNA library. For each gene, a non-template control (NTC) was included. The following thermal cycling protocol was followed: denaturation at 95°C for three minutes, followed by 40 cycles of denaturation at 95°C for 25 seconds, annealing at 60°C for 25 seconds and extension at 72°C for 15 seconds. Data collection was carried out in the elongation phase. For melting curve analysis, the temperature was increased from 60°C to 95°C at 0.5 degree increments every 5 seconds. All data analysis was carried out using CFX Maestro® software.

The selection of five genes for RT-qPCR analysis was based on their significant up or down-regulation in the controls vs cases at T1 comparison (*G0S2* and *AREG* up-regulated in cases), or the cases at T1 vs cases at T2 comparison (*PTGDR2* and *IL5RA* up-regulated at sampling time 2*, G0S2* and *BMX* down-regulated at sampling time 2). Expression patterns of the five selected transcripts matched the direction of expression observed from the RNA-Seq data (**supplementary** **table** **8**).

| **Target** | **Biological Group** | **Relative Normalised Expression** | **Expression 95% CI Low** | **Expression 95% CI High** | **P-Value** |
| --- | --- | --- | --- | --- | --- |
| *AREG* | Cases at sampling time 1 vs Controls at sampling time 1 | 3.72 | 2.18 | 6.33 | 0.03 |
| *BMX* | Cases at sampling time 1 vs Cases at sampling time 2 | 3.53 | 1.86 | 6.69 | <0.01 |
| *IL5RA* | Cases at sampling time 1 vs Cases at sampling time 2 | 0.19 | 0.08 | 0.42 | <0.01 |
| *GOS2* | Cases at sampling time 1 vs Cases at sampling time 2 | 0.43 | 0.25 | 0.72 | 0.05 |
| *PTGDR2* | Cases at sampling time 1 vs Cases at sampling time 2 | 2.89 | 1.95 | 4.26 | 0.01 |

**Supplementary table 8**

**qPCR expression data derived using 2^ΔΔCt^ method** **for selected transcripts *AREG, BMX, G0S2, IL5RA and PTGDR_2_,* normalised to *GAPDH* (glyceraldehyde-3-phosphate dehydrogenase) as a housekeeping gene. *P*-values were computed using the ANOVA (one-way analysis of variance) test and Tukey’s post hoc test.**

**Supplementary table 9**

| **Classification Group** | **Percentage (%)** | **Minutes** |
| --- | --- | --- |
| 1. ***Evolution:*** |  |  |
| Recovery | 86 | N/A |
| Static | 0 | N/A |
| Relapsing | 14 | N/A |
| 1. ***Progressive:*** |  |  |
| Yes | 14 | N/A |
| No | 86 | N/A |
| 1. ***Organ System:*** |  |  |
| Neurological | 29 | N/A |
| Cardiopulmonary | 29 | N/A |
| Limb pain exclusively | 0 | N/A |
| Skin | 100 | N/A |
| Lymphatic | 14 | N/A |
| Vestibular | 14 | N/A |
| 1. ***Time of onset:*** |  |  |
| Time before surfacing | N/A | 0* |
| Time after surfacing | N/A | 43 (mean) |
| 1. ***Gas Burden:*** |  |  |
| Low | 43 | N/A |
| Medium | 57 | N/A |
| High | 0 | N/A |
| 1. ***Evidence of Barotrauma:*** |  |  |
| Pulmonary Barotrauma | 0 | N/A |
| Ear Barotrauma | 0 | N/A |
| Sinus Barotrauma | 0 | N/A |

**Francis and Smith classification of dysbaric illness manifestations in the case group**
